# Evolution-Based Protein Engineering for Antifungal Peptide Improvement

**DOI:** 10.1093/molbev/msab224

**Published:** 2021-07-28

**Authors:** Jing Gu, Noriyoshi Isozumi, Shouli Yuan, Ling Jin, Bin Gao, Shinya Ohki, Shunyi Zhu

**Affiliations:** 1 Group of Peptide Biology and Evolution, State Key Laboratory of Integrated Management of Pest Insects and Rodents, Institute of Zoology, Chinese Academy of Sciences, Beijing, China; 2 University of Chinese Academy of Sciences, Beijing, China; 3 Center for Nano Materials and Technology (CNMT), Japan Advanced Institute of Science and Technology (JAIST), Ishikawa, Japan

**Keywords:** antimicrobial peptide, *Candida albicans*, Cremycin-5, epistasis, paralog, universally enhanceable activity-modulating site (UEAMS)

## Abstract

Antimicrobial peptides (AMPs) have been considered as the alternatives to antibiotics because of their less susceptibility to microbial resistance. However, compared with conventional antibiotics they show relatively low activity and the consequent high cost and nonspecific cytotoxicity, hindering their clinical application. What’s more, engineering of AMPs is a great challenge due to the inherent complexity in their sequence, structure, and function relationships. Here, we report an evolution-based strategy for improving the antifungal activity of a nematode-sourced defensin (Cremycin-5). This strategy utilizes a sequence-activity comparison between Cremycin-5 and its functionally diverged paralogs to identify sites associated with antifungal activity for screening of enhanceable activity-modulating sites for subsequent saturation mutagenesis. Using this strategy, we identified a site (Glu-15) whose mutations with nearly all other types of amino acids resulted in a universally enhanced activity against multiple fungal species, which is thereby defined as a Universally Enhanceable Activity-Modulating Site (UEAMS). Especially, Glu15Lys even exhibited >9-fold increased fungicidal potency against several clinical isolates of *Candida albicans* through inhibiting cytokinesis. This mutant showed high thermal and serum stability and quicker killing kinetics than clotrimazole without detectable hemolysis. Molecular dynamic simulations suggest that the mutations at the UEAMS likely limit the conformational flexibility of a distant functional residue via allostery, enabling a better peptide–fungus interaction. Further sequence, structural, and mutational analyses of the Cremycin-5 ortholog uncover an epistatic interaction between the UEAMS and another site that may constrain its evolution. Our work lights one new road to success of engineering AMP drug leads.

## Introduction

Antibiotic resistance has become a severe threat to public health over the past few decades, and what’s more, the development of new antibiotics lags behind the emergence of resistance (Neu 1992; Högberg et al. 2010). Antimicrobial peptides (AMPs, a generic term for antibacterial, antifungal, antiviral, and antimalarial peptides) show a broad-spectrum antibiotic activity against bacteria, fungi, viruses, and malaria with less susceptibility to microbial resistance and thus have been considered as a class of promising candidates for developing novel antibiotics (Zasloff 2002; Brogden 2005; Hancock and Sahl 2006; Peschel and Sahl 2006; Fox 2013). Nevertheless, these naturally produced peptides must be optimized to overcome their intrinsic limitations, especially suboptimal efficacy and the consequent high cost and nonspecific cytotoxicity (Marr et al. 2006; Vaara 2009). Considerable efforts have been made to develop strategies with an aim to improve their efficacy through adjusting charge and amphiphilicity, creating “linguistic model,” exploiting quantitative structure-activity relationship (QSAR), and developing high throughput screening (HTS) systems to specifically address optimization of AMPs (Raventós et al. 2005; Loose et al. 2006; Cherkasov et al. 2009; Fjell et al. 2011; Wiradharma et al. 2013; Ong et al. 2014; da Cunha et al. 2017). Although these traditional engineering technologies have already achieved some success, it should be noted that they may be limited by the inherent complexity of AMPs in sequence–structure–function relationships and the difficulty in antimicrobial assays adapted for HTS as well as the requirements of expensive resources and person hours (Swint-Kruse 2016). Thus, new strategies are needed to overcome the limitations of the initial engineering technologies.

**Fig. 1.**
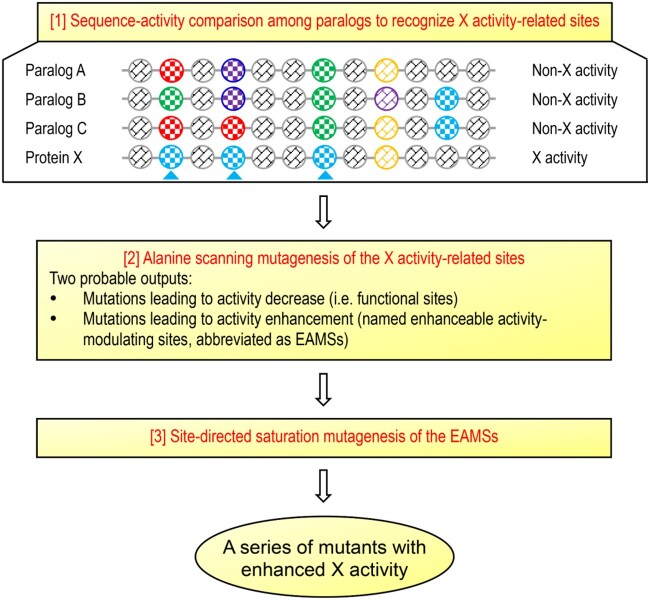
Schematic diagram of the paralog-based discovery of EAMSs for SM. Assuming that protein X with X activity has three paralogs (*A*, *B*, and *C*) that all lose the activity during evolution, one can rapidly identify the X activity-related amino acid sites in protein X (herein marked by *cyan* triangles). Alanine scanning mutagenesis of such sites will probably generate two mutational outputs, from which the EAMSs will be used for subsequent SM to create a series of mutants with improved functional property. Scribed circles denote amino acids whose side chains are distinguished by different colors.

From an evolutionary perspective, a protein comprises conserved and nonconserved sites. Mutagenesis of individual conserved sites is often catastrophic due to their key roles in maintaining protein architecture and fast folding and the interactions with other molecules (i.e., biological function) (Worth et al. 2009; Swint-Kruse 2016). By contrast, nonconserved sites can often tolerate multiple mutations without disrupting the overall structure of a protein and is thus a candidate target of engineering to fine-tune protein functional parameters (Swint-Kruse 2016). However, these sites occupy the majority of a protein, screening of functionally enhanceable mutants from them requires a deep mutational scanning of each such site to record mutational effect (Fowler and Fields 2014), limiting its application in AMP engineering. As an alternative, we introduced an evolutionary strategy based on sequence-activity comparison among AMP paralogs to rapidly identify antimicrobial activity-associated nonconserved sites for activity improvement by site-directed saturation mutagenesis (SM) (fig. 1), thus reducing the need for extensive mutagenesis. The rationality behind this strategy is that many duplicated genes (paralogs) have undergone functional divergence through mutations in their open reading frames (Presgraves 2005), which allows one to establish a correlation between their sequences (amino acid sites) and the activity (fig. 1). Because these sites are closely associated with the activity, it is logical to infer two probable outputs when mutated: 1) Mutations leading to activity decrease indicating functional sites; and 2) mutations leading to activity enhancement indicating enhanceable activity-modulating sites (EAMSs). We tested the feasibility of this strategy in improving Cremycin-5 (abbreviated as Crem-5 in place of initial “Crm-5” for better English pronunciation), a fruit nematode-derived antifungal peptide of 42 residues belonging to the Drosomycin (Drs)-type defensin family (Zhu and Gao 2014). This family of members is extensively present in plants and some ecdysozoans. Different from Drs whose activity is mainly limited to filamentous fungi (Fehlbaum et al. 1994), Crem-5 exhibits an attractive activity on the yeast pathogen *Candida albicans* (Zhu and Gao 2014), and was thus chosen in this work.

Through comparing the sequence-antifungal activity relationship between Crem-5 and its paralogs in combination with mutagenesis experiments, we identified four Crem-5-specific, functionally important sites and one EAMS in Crem-5. Further SM of the EAMS with nearly all other types of amino acids created a series of Crem-5 mutants with a remarkably enhanced antifungal activity, corroborating the feasibility of our strategy. A mutant (Glu15Lys) exhibiting the most enhanced activity was further assayed in terms of its mode of action and therapeutic potential toward *C. albicans* B16. Furthermore, we found that such an unusual mutational output could be probably a consequence of remote mutation-elicited allosteric effect changing the conformational dynamics of functional sites. Finally, we provide evidence for epistatic interaction between the EAMS and another site that might constrain the evolution of the former.

## Results

### Crem-5 Containing One EAMS

**Fig. 2.**
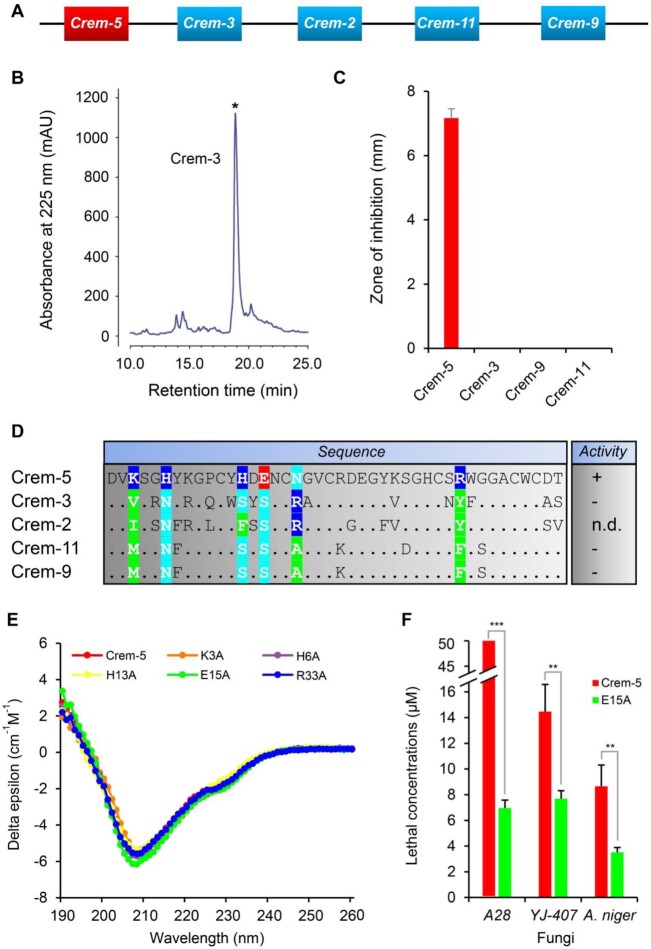
Identification of EAMSs from the antifungal activity-related sites in Crem-5. (*A*) Chromosomal location of Crem-5 (in red) and its four paralogs (in cyan), which was redrawn from Zhu and Gao (2014). (*B*) RP-HPLC profile of recombinant Crem-3 (as a representative). The product collected for analysis is marked by an asterisk. (*C*) Comparison of antifungal activity of Crem-5 with that of its paralogs against *Aspergillus niger*. Peptide amount used in each well is 1.0 nmol. Inhibition zone diameters are mean±SD (*n* = 3). For other fungi, the results were similar. (*D*) The identification of antifungal activity-related sites by sequence-activity comparison. Identical amino acid residues to Crem-5 are denoted as dots. Crem-5-specific sites are shaded in color according to their side-chain nature (green, hydrophobic and aromatic; cyan, polar; blue, basic; and red, acidic). n.d., not determined; +, active; −, inactive. (*E*) CD spectra of Crem-5 and its five mutants. (*F*) Comparison of *C*_L_ between Crem-5 and E15A against three species of fungi. The larger the value, the weaker the antifungal activity. A28, *A. nidulans* A28; YJ-407, *A. fumigatus* YJ-407. Data are presented as mean±SD (*n* = 3). *P* values obtained by two-tailed Student’s *t*-test.

According to the new strategy (fig. 1), we firstly compared the antifungal activity of Crem-5 with that of the three paralogs (Crem-3, -9, and -11) (fig. 2*A*) to identify its antifungal activity-related sites by using purified recombinant peptides expressed in *Escherichia coli* (fig. 2*B* and supplementary fig. S1*A*, Supplementary Material online). Their encoding genes are tandemly arranged on chromosome (fig. 2*A*) and have been previously proved to be transcriptionally active (Zhu and Gao 2014). We showed that all paralogs except Crem-5 exhibited no antifungal activity against *Aspergillus niger* (fig. 2*C*), allowing rapid recognition of six Crem-5-specific sites associated with the activity, including K^3^, H^6^, H^13^, E^15^, N^18^, and R^33^ (fig. 2*D*).

To identify possible EAMSs from the six sites, we utilized alanine scanning mutagenesis (Cunningham and Wells 1989) to prepare all alanine mutants of these sites except N^18^ (supplementary table S1, Supplementary Material online) and compared their structures and activity with those of the unmodified peptide against three species of fungi (*A. nidulans* A28, *A. fumigatus* YJ-407, and *A. niger*). We found that alanine mutations at these nonconserved sites caused no obvious structural change, as identified by their highly similar CD spectra (fig. 2*E*). However, individual mutations of the four positively charged residues (K3A, H6A, H13A, and R33A) resulted in the complete loss or a remarkable reduction in activity (supplementary table S2, Supplementary Material online). In contrast, E15A did obtain a remarkably enhanced fungicidal activity compared with the wild-type peptide toward all the three fungi (fig. 2*F*). These results highlight the functional importance of the four cationic residues in conferring the fungicidal activity of Crem-5 and especially the acidic E^15^ as an EAMS, which is fully in line with our prediction (fig. 1). The functional role of cationic amino acids has been documented previously in Drs (Zhang and Zhu 2010), but mutations of an acidic amino acid in these two antifungal defensins produced two quite opposite consequences (decrease in Drs but increase in Crem-5).

### General Functional Enhancement in Glu-15 Saturation Mutants

To explore the potential of E^15^ as an engineering target by SM, we individually introduced other 18 different amino acids into this site through inverse PCR (supplementary table S3, Supplementary Material online). Cysteine was not included in this design in view of its key role in disulfide bridge formation. Among these mutants, 15 were successfully expressed and purified to homogeneity, and three (E15L, E15G, and E15W) were not obtained due to nonspecific cleavage of the fusion proteins or the formation of insoluble inclusion bodies (supplementary table S1, Supplementary Material online). Again, all the mutants displayed similar CD spectra to Crem-5 (supplementary fig. S1*B*, Supplementary Material online), indicative of no significant structural alteration by SM. Using two representative filamentous fungi (*A. niger* and *A. nidulans*) and four clinical isolates of *C. albicans* (strains 2.4138, B16, GD18, and S068) (supplementary table S4, Supplementary Material online) as test fungi, we evaluated the fungicidal activity of these recombinant peptides using the inhibition-zone method to determine their lethal concentrations (C_L_) (supplementary fig. S2, Supplementary Material online). Of the 15 mutants, 14 showed ≥1-fold higher activity on three *C. albicans* strains (B16, GD18, and S068), 13 on *A. nidulans* A28, 10 on *C. albicans* 2.4138, and 7 on *A. niger* (table 1). Such a differential response likely reflects the difference in the cellular structures or components among these fungal strains. The only one that did not improve the activity on any strains used here was the conservative replacement (E15D). Of the 14 mutants, 43% (i.e., E15H, E15K, E15M, E15N, E15R, and E15Y) exhibited greater activity than the parental peptide against all the six strains, 36% (i.e., E15A, E15F, E15Q, E15S, and E15T) against five of the six strains, 14% (i.e., E15I and E15V) against four of the six strains, and 7% (i.e., E15P) against three of the six trains. Of the mutants that owned the most wide-spectrum activity, most obtained >4-fold increased activity and particularly E15K acquired the strongest potency against five fungal strains, showing a 9.5- to 15.8-fold increase (table 1).

**Table 1.** Lethal Concentrations (C_L_, μM) of Crem-5 and Its Mutants against Fungi.

	*Aspergillus niger*	*Aspergillus nidulans* A28	*CA* 2.4138		*CA* B16	*CA* GD18	*CA* S068
Crem-5	4.03	W.A.	57.96		35.05	57.96	89.62
E15A	2.74	7.25 (+>5.9)	15.52 (+2.7)		8.93 (+2.9)	12.45 (+3.7)	15.16 (+4.9)
E15D	5.07	W.A.	77.98		44.69	44.70	71.63
E15F	0.87 (+3.6)	10.98 (+>3.6)	47.60		7.64 (+3.6)	19.85 (+1.9)	17.22 (+4.2)
E15H	0.94 (+3.3)	8.87 (+>4.6)	7.93 (+6.3)		5.02 (+6.0)	7.60 (+6.6)	9.55 (+8.4)
E15I	2.11	14.08 (+>2.6)	35.26		15.16 (+1.3)	24.46 (+1.4)	26.75 (+2.4)
E15K	1.35 (+2.0)	4.26 (+>10.7)	5.35 (+9.8)		3.35 (+9.5)	5.35 (+9.8)	5.35 (+15.8)
E15K/G36A	7.58	17.91	51.21		28.11	44.99	68.23
E15M	1.31 (+2.1)	4.39 (+>10.4)	8.06 (+6.2)		3.54 (+8.9)	7.90 (+6.3)	8.37 (+9.7)
E15N	1.58 (+1.6)	7.05 (+>6.1)	12.45 (+3.7)		6.24 (+4.6)	10.58 (+4.5)	8.06 (+10.1)
E15P	2.69	W.A.	39.60		16.87 (+1.1)	28.75 (+1.0)	35.17 (+1.5)
E15Q	2.39	10.85 (+>3.6)	21.39 (+1.7)		11.29 (+2.1)	21.39 (+1.7)	19.59 (+3.6)
E15R	0.70 (+4.7)	6.24 (+>7)	8.85 (+5.5)		8.06 (+3.3)	6.88 (+7.4)	7.58 (+10.8)
E15S	3.33	8.59 (+>4.8)	17.86 (+2.2)		7.29 (+3.8)	21.83 (+1.7)	21.27 (+3.2)
E15T	3.52	7.85 (+>5.4)	18.59 (+2.1)		8.24 (+3.3)	14.46 (+3.0)	15.52 (+4.8)
E15V	2.92	8.84 (+>4.7)	53.50		17.86 (+1.0)	29.39 (+1.0)	28.75 (+2.1)
E15Y	1.77 (+1.3)	7.23 (+>5.9)	16.78 (+2.5)		7.93 (+3.4)	11.49 (+4.0)	12.65 (+6.1)

Note.—Fold increase is calculated as (*C*_L_ of Crem-5−*C*_L_ of a mutant)/*C*_L_ of a mutant, in which the *C*_L_ of Crem-5 on *A. nidulans* A28 was treated as >50 μM. Only >1-fold increase is marked by plus sign.

CA, *Candida albicans*; W.A., weak activity, indicating that only very small inhibition zones were observed at 2.0 nmol peptide/well.

**Fig. 3.**
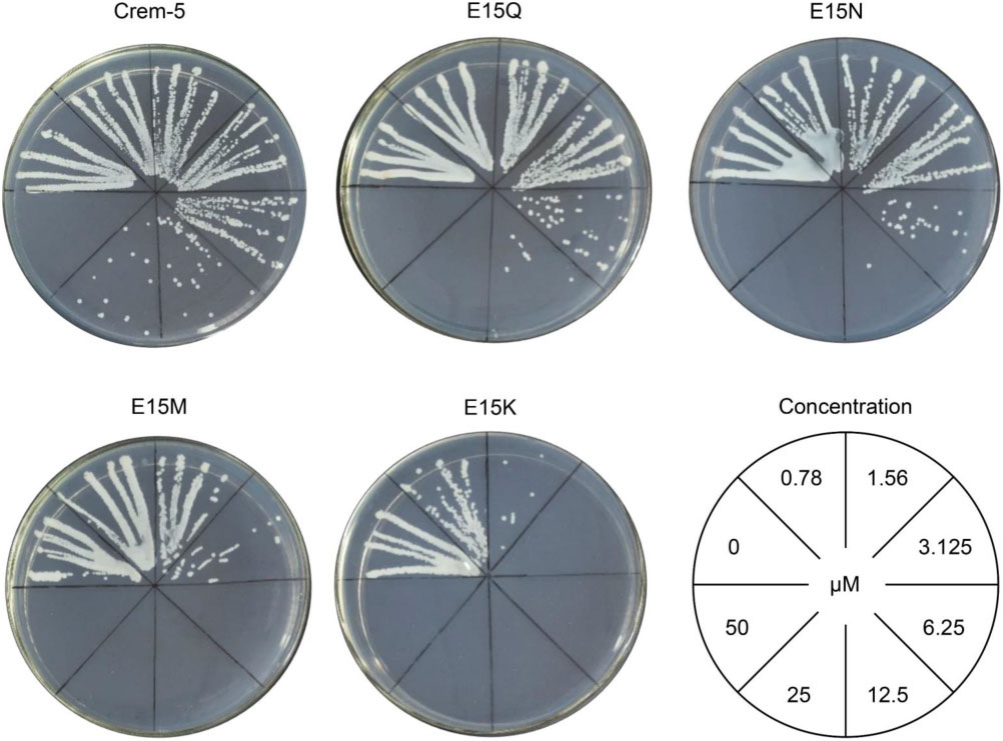
MICs of Crem-5 and representative mutants. *Candida albicans* B16 incubated overnight with various concentrations of Crem-5, E15Q, E15N, E15M, or E15K were streaked on agar plates. The figure is representative of three independent experiments.

To validate the *C*_L_ determinations on agar plates, we employed liquid inhibition assay to evaluate the activity of Crem-5 together with its four representative mutants (E15K, E15M, E15N, and E15Q) against *C. albicans* B16, a pathogen yeast isolated from the vagina of Chinese women suffering from vulvovaginal candidiasis (Ge et al. 2012). After treated overnight by the peptides at varying concentrations, the B16 cultures were streaked on agar plates to observe the cell viability (fig. 3). The minimum inhibitory concentrations (MICs) based on this assay were 50 μM for Crem-5; 25 μM for E15Q; 25 μM for E15N; 6.25 μM for E15M; and 3.125 μM for E15K, which were by and large consistent with their *C*_L_ (table 1). In this experiment, the mutations increased the activity of Crem-5 by 1-, 1-, 7-, and 15-fold, respectively (fig. 3).

Overall, our data reveal that the universality of the E^15^ mutation-mediated functional enhancement occurs at both the fungal targets and the side-chain chemistry of the peptide.

### Fungicidal Properties and Therapeutic Potential of E15K

**Fig. 4.**
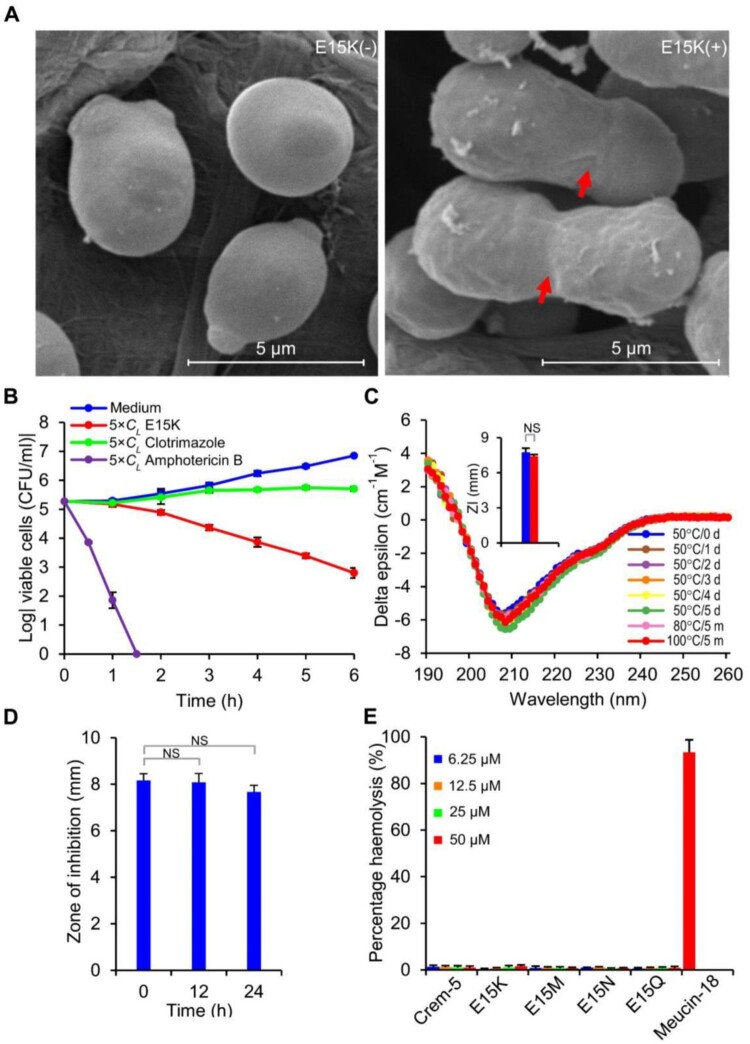
Effects of E15K on *Candida albicans* B16 and its therapeutic potential. (*A*) Scanning electron microscopic observation of *C. albicans* B16 in the absence or presence of E15K. The red arrow indicates cells with a failed cytokinesis. (*B*) Time killing kinetics of E15K. Clotrimazole, an antifungal medication that inhibits the synthesis of ergosterol (a component of fungal cell membranes), resulting in increased cellular permeability (Warrilow et al. 2010); and amphotericin B, an antifungal medication that binds with ergosterol, forming pores that cause rapid leakage of monovalent ions (Mesa-Arango et al. 2012), were used as control. (*C*) Thermal stability of E15K, evaluated by CD spectral analysis of heat-treated peptides. Inset, residual fungicidal activity, indicated by zones of inhibition (ZI) formed by 1.0 nmol of peptides, after incubation at 100 °C for 5 min (blue, no heating; red, heating. *n* = 3). (*D*) Serum stability of E15K (1.0 nmol). (*E*) Hemolytic effect of Crem-5, E15K, E15M, E15N, and E15Q on mouse red blood cells. Meucin-18 (Gao et al. 2009) was used as control. (*B*–*E*) Data are mean±SD (*n* = 3). (*C* and *D*) *P* values obtained by two-tailed Student’s *t*-test. NS, not significant.

Based on the enhanced activity, we undertook new studies to assess the fungicidal properties and the therapeutic potential of E15K on *C. albicans* B16. Scanning electron microscopic (SEM) observation of the E15K-treated cells revealed that this peptide, like Crem-5, inhibited the cytokinesis (fig. 4*A* and supplementary fig. S3, Supplementary Material online). Without the peptide, *C. albicans* cells divided normally with bud scars on the smooth cellular surface whereas the treated cells were not capable of dividing into two single daughter cells and their cellular surface became rougher, indicating that the mutation does not alter the killing mechanism (Zhu and Gao 2014). Time killing kinetics showed that E15K killed *C. albicans* B16 cells in a rate falling in between the metabolic inhibitor—clotrimazole and the pore former—amphotericin B (fig. 4*B*). When E15K was incubated in water at 50 °C for 1–5 days or 80–100 °C for 5 min, its activity and structure were not obviously changed (fig. 4*C*), demonstrating its high thermal stability. When incubated in the mouse serum exceeding 24 h, the peptide retained its complete antifungal activity, demonstrating its serum stability (fig. 4*D*). E15K caused no detectable hemolysis on mouse erythrocytes at 50 μM (fig. 4*E*).

### Evidence for Allosteric Communication between EAMS and Functional Site

**Fig. 5.**
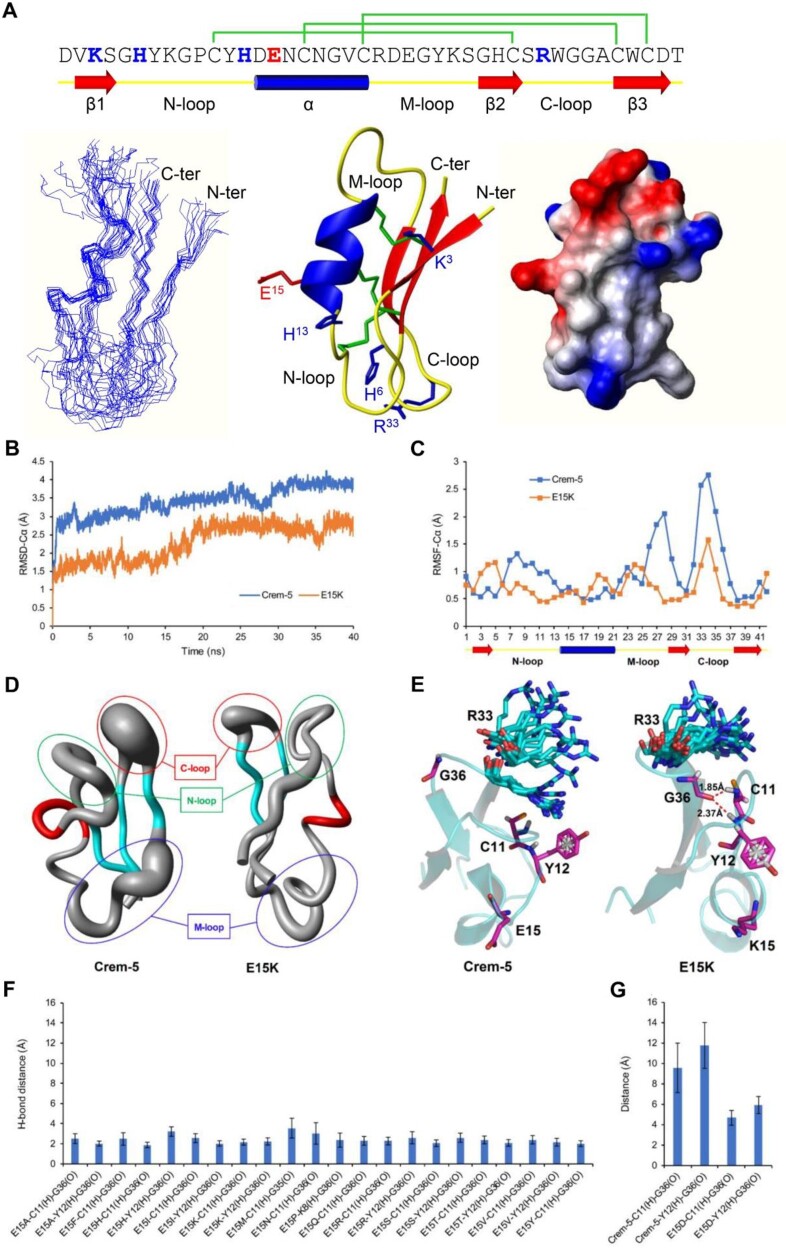
3D structure of Crem-5 and MD simulations of Crem-5 and its mutants. (*A*) The structure of Crem-5. The disulfide bridge connectivity pattern and secondary structure elements (cylinder: α-helix; arrow: β-strand) (top) are extracted from its NMR structure. Functional residues and the EAMS are shown in blue and red, respectively. The amino-, middle-, and carboxyl-terminal loops are designated as N-, M-, and C-loop, respectively. Left, a family of 15 lowest energy structures superimposed over the backbone atoms. Middle, a ribbon model with disulfide bridges (green), functional residues (blue), and the EAMS (red) shown in stick mode. Right, surface potential distribution with negative (red), positive (blue), and neutral (white) charge zones. Structurally, the EAMS is located on the α-helix and the functional residues are situated on the N-loop (H^6^ and H^13^) and its vicinity (K^3^) along with the C-loop (R^33^). Most of them appear in one end of the rectangle molecule. (*B*) RMSD-Cα of Crem-5 and E15K in 40 ns simulation. (*C*) RMSF-Cα of Crem-5 and E15K (20–40 ns). (*D*) A “sausage” model displaying the conformational flexibility of the two molecules based on 2,001 dynamic conformers (20–40 ns). (*E*) Overlay of 20 snapshots obtained from 20 to 40 ns trajectories (take one every 1 ns). C^11^/Y^12^, and G^36^ are represented in magenta and R^33^ in cyan. The distances of hydrogen bonds between C^11^, Y^12^, and G^36^ are displayed according to the last conformation of E15K. (*F*) Average distances of H-bonds (20–40 ns) between C^11^/Y^12^ and G^36^ in the mutants with enhanced activity. (*G*) Average distances between C^11^/Y^12^ and G^36^ in Crem-5 and E15D (20–40 ns). Data are expressed as mean±SD (*n* = 2,001).

Prompted by the observation that SM at the EAMS induced a general functional enhancement in Crem-5, we tried to explore how the mutations affect the dynamics of functional residues using molecular dynamics (MD) simulations. To this end, we firstly determined the experimental structure of Crem-5 using nuclear magnetic resonance (NMR) spectroscopy analysis of a ^15^N-labeled protein (supplementary table S5, Supplementary Material online). The resulting family of 20 structures is shown in figure 5*A* (left), the final average structure obtained by molecular dynamic simulation in figure 5*A* (middle), and the molecular surface with electrostatic potential in figure 5*A* (right). Similar to Drs, Crem-5 adopts a typical cysteine-stabilized α-helical β-sheet (CSαβ) fold comprising an α-helix and a three-stranded antiparallel β-sheet stabilized by three disulfide bridges (Cys11–Cys31, Cys17–Cys38, Cys21–Cys40) (fig. 5*A*).

Next, we conducted 40 ns of MD simulations of Crem-5 and its E^15^ mutants using the GROMACS software package. As shown in figure 5*B*, E15K showed a smaller root mean square deviation (RMSD) in its Cα atoms than Crem-5 during simulations, suggesting that this mutation overall increased the stability of the peptide. Further analyses of the root mean square fluctuation (RMSF) of Cα atoms and a “sausage” model generated from 2,001 dynamic conformers identified the rigidity-enhanced regions mainly occurring in the three loops (labeled N-, M-, and C-loop) (fig. 5*C*and *D*). In E15K, two strong hydrogen bonds (H-bonds) are formed between G^36^ (C-loop) and C^11^/Y^12^ (N-loop) with a distance of 1.85–2.37 Å (fig. 5*E*). This could restrict the conformational flexibility of the glycine, leading to an increased rigidity of its adjacent functional residue R^33^ (fig. 5*E*), thereby strengthening the interactions between the peptide and fungi. For all other enhanced mutants there also exists exclusively at least one of the two H-bonds whereas for Crem-5 and E15D they both are absent (>4 Å) (fig. 5*F* and *G*). This kind of presence pattern further consolidates the role of G^36^ in conferring to these mutants an enhanced activity through H-bond formation to transfer the mutational signal from sites 15 to 33 with the help of C^11^ and Y^12^ (fig. 5*E*). When destructing the H-bonds to cut off the pathway by the mutation G36A in E15K, we found that the overall structure of the mutant was destroyed, as reflected by a significant blue shift in its CD spectra (supplementary fig. S1*B*, Supplementary Material online), in line with its decreased activity (table 1). This experiment reveals the structural importance of G^36^ in maintaining both global folding and mutant-dependent local H-bond formation. Based on these data, we assumed that SM of the EAMS might serve as a common command to allosterically regulate the conformational dynamics of remote functional residues, consequently eliciting a universal activity enhancement. A similar case might also occur in human angiotensin converting enzyme 2 (ACE2), in which nearly all substitutions at Asn-90 or Thr-92, both distal from the interface (Wang et al. 2020), are highly favorable for RBD binding (Chan et al. 2020), suggesting a common manner of mutation-elicited allosteric regulation. In this case, introduction of more mutations at an EAMS by nonnatural amino acids (e.g., D-amino acids and amino acid analogs) or even chemical modifications may be beneficial but still awaits further investigation.

### Epistatic Interaction Limiting the Evolution of EAMS

**Fig. 6.**
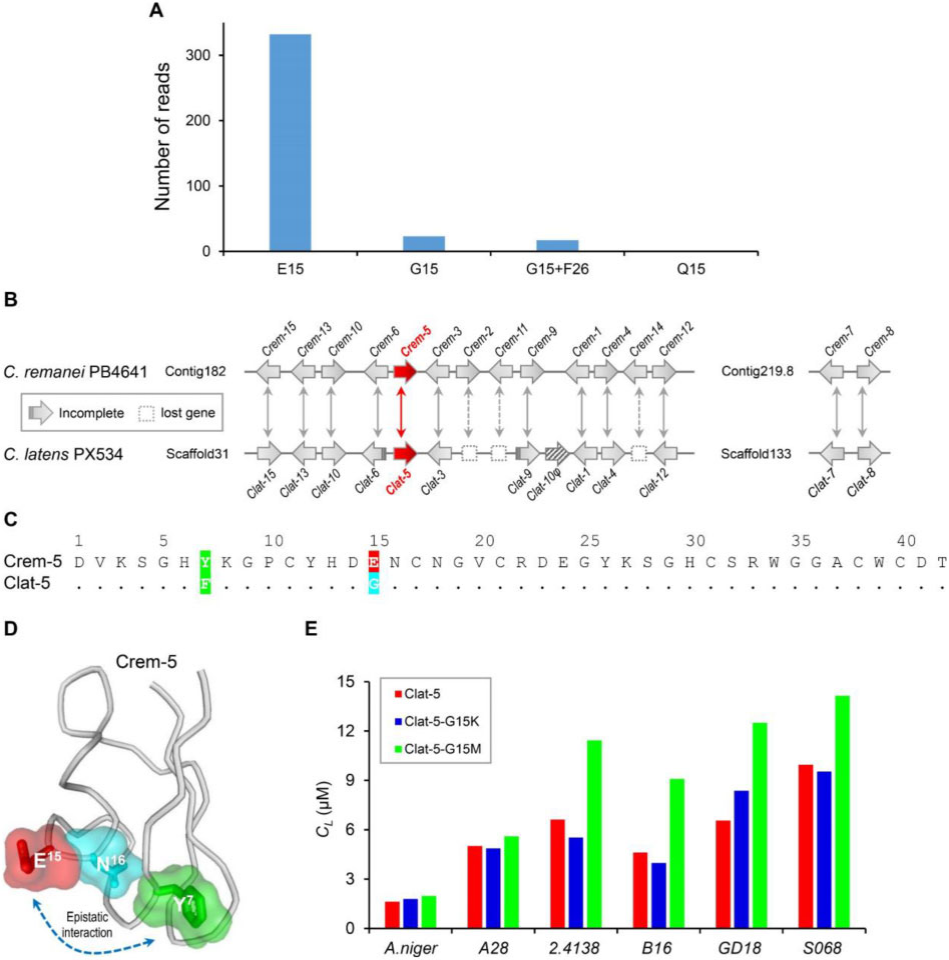
Evidence for epistatic interaction between sites 7 and 15 of Crem-5. (*A*) The polymorphism of *Crem-5* in *Caenorhabditis remanei*. The reads were retrieved by BlastN search of the NCBI SRA database with *Crem-5* as query and their numbers were counted according to the polymorphism of site 15 that included E, G, and Q. In 17 out of 23 reads that contain a G at site 15, a Y to F mutation at site 26 co-occurs. Raw sequence reads are provided in appendix 1. (*B*) Synteny mapping of *crem* and *clat* genes in *Cae. remanei* PB4641 and *Cae. latens* PX534. Arrow directions refer to the open reading frame orientation of genes. The arrow filled with diagonals marks the pseudogene of a *Clat-10* duplicate and virtual end arrows indicate incomplete sequences. Double arrows indicate orthologous relationship between Crems and Clats where Crem-5 and its orthologous Clat-5 are arrowed in red. Lost genes in *Cae. latens* are denoted with dotted boxes. (*C*) Sequence comparison between Crem-5 and Clat-5. Identical amino acid residues to Crem-5 are denoted as dots in Clat-5 and different residues are shaded in color according to their chemical features (green, aromatic; cyan, polar; and red, acidic). (*D*) Epistatic interactions between sites 7 and 15 are mediated by their adjacent site 16. The structure displayed here is the last MD simulation frame. (*E*) Comparison of C_L_ values between Clat-5 and its mutants against six strains of fungi. The larger the value, the weaker the antifungal activity. A28, *Aspergillus nidulans* A28; 2.4138, *Candida albicans* 2.4138; B16, *C. albicans* B16; GD18, *C. albicans* GD18; S068, *C. albicans* S068.

To understand the reason why evolution chose E^15^, a residue that only confers the lowest antifungal activity of Crem-5 among nearly all possible amino acids, we firstly undertook the Sequence Read Archive (SRA) database search to obtain polymorphism data of *Crem-5* from *Caenorhabditis remanei*. The data show that E^15^ occurs in a rather high frequency (>93%) whereas two other amino acids (G and Q) at this position only have <7% of frequency (fig. 6*A*), indicating that this acidic residue is highly selected by evolution. Because in most reads containing a G at site 15, a Y to F mutation at site 26 co-occurs, it is reasonable to suppose that the fixation of E^15^ in Crem-5 is a consequence of epistasis, a prevailed force that determines the likelihood of fixation of an amino acid change through evolution (Miton and Tokuriki 2016; Poelwijk 2019). To provide support for this supposition, we performed an ortholog comparison by building the synteny map of *Crem* genes in *Cae. remanei* PB4641 and homologous genes in *Cae. latens* PX534 (herein named *clatencin* genes, abbreviated as *Clat*) to define the ortholog of Crem-5 (i.e., Clat-5) (fig. 6*B* and supplementary table S6, Supplementary Material online). As shown in figure 6*C*, Crem-5 and Clat-5 differ by two substitutions (E15G and Y7F), which is similar to the polymorphism data (fig. 6*A*), suggesting that the fixation of a non-Glu amino acid at site 15 depends on a change at site 7. Such a dependence can be explained by their positions in the structure where E^15^ and Y^7^ mutually interact with Asn^16^ (N^16^) (fig. 6*D*).

Since the context-dependent mutation effect between two orthologs is the hallmark of epistasis (Poelwijk 2019), we tested this behavior in the two nematode defensins by using recombinant peptides, in which two mutations were introduced at site 15 (supplementary fig. S4*A* and *B*, Supplementary Material online). We found that all mutants retained a parent structure except Clat-5-G15K that had a slightly changed structure, as identified by a minor blue shift in its CD spectra (supplementary fig. S4*C*, Supplementary Material online). Different from the Crem-5 mutants E15K and E15M that both were more active than their parent peptide, the Clat-5 mutants G15K and G15M maintained or decreased their parent peptide activity (table 1 and fig. 6*E*). Such a difference clearly reflects their genetic background differential, that is, Y or F at site 7. The epistatic interaction between sites 7 and 15 is further strengthened by their impact on protein folding since the mutant E15G in Crem-5 was expressed as an insoluble form (inclusion body) in *E. coli* but a compensatory mutation (Y7F), as in the case of Clat-5, restored the soluble expression. These observations could suggest a putative pathway in the evolution of the *Caenorhabditis* defensins, that is, Crem-5 (Y^7^/E^15^) → F^7^/E^15^ → Clat-5 (F^7^/G^15^).

## Discussion

As the most common opportunistic human fungal pathogen, *C. albicans* causes both superficial and systemic infections of high-risk individuals with immunologic defects (Poulain 2015). Many of the *Candida* species, including *C. albicans*, have developed resistance worldwide to azoles and echinocandins, two antifungal drugs used globally to treat *Candida* infections, posing a serious public health threat (Pristov and Ghannoum 2019). This work reports a successful case of AMP engineering based on a paralog sequence-activity comparison combined with site-directed SM. Using this strategy, we created a batch of new leads for developing drugs treating candidiasis, as exemplified by most Crem-5 mutants showing enhanced activity against the fungal pathogen *C. albicans* B16 in low micromolar concentrations.

**Fig. 7.**
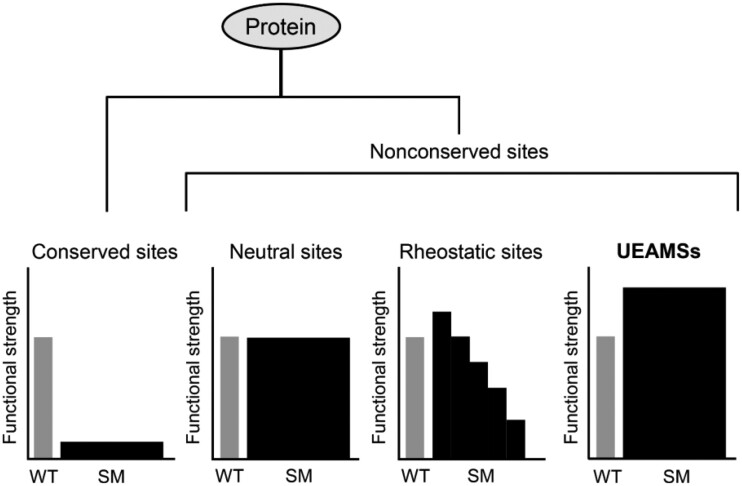
Partitioning protein sites by SM effects on function. In this classification, SM at a conserved site will be probably deleterious to the protein, whereas SM at a nonconserved site will cause three different outputs: 1) If all variants from a nonconserved site possess a similar function to the wild-type protein, it is called a neutral site (Swint-Kruse 2016). 2) If mutational outputs range progressively over orders of magnitude, it is called a rheostatic site (McLaughlin et al. 2012; Meinhardt et al. 2013; Ishwar et al. 2015; Swint-Kruse 2016; Hodges et al. 2018). 3) If nearly all substitutions exhibit a universally enhanceable function, it is called a UEAMS (this work).

As mentioned previously, mutations at conserved sites often impair the structure or/and function of a protein. However, extensive studies have seen different outputs elicited by mutations at nonconserved sites (fig. 7). For example, their mutants produced by any amino acid replacements function like a wild-type protein, which are called neutral sites (Swint-Kruse 2016). Some other nonconserved sites exhibit a rheostat mutational behavior, as identified by mutations with different amino acids leading to a range of functional outcomes (called rheostatic site) (McLaughlin et al. 2012; Meinhardt et al. 2013; Ishwar et al. 2015; Swint-Kruse 2016; Hodges et al. 2018). Our study demonstrates that there may exist a third class of nonconserved sites (e.g., E^15^ in Crem-5) whose mutations by most replacements can cause a universally enhanced activity. They are present in the nonconserved sites associated with a specific biological activity and thus can be quickly identified by paralog sequence-activity comparison. Their prevalence is evidenced by a survey of publications that sees more such examples in several biologically diverse proteins: 1) Human antibody (IgG1). Mutations of its Lys^326^ (K^326^) (lysine at site 326) with Ala (A), Arg (R), Asn (N), Asp (D), Gln (Q), Glu (E), Gly (G), His (H), Met (M), Phe (F), Trp (W), Tyr (Y), and Val (V) all increased C1q binding (Idusogie et al. 2001). 2) Bovine pancreatic phospholipase A2 (PLA2). Mutations of its K^56^ with N, E, Ile (I), M, F, and Thr (T) all increased the catalytic activity by 2- to 5-folds (Noel et al. 1991). 3) Fungal defensin-like peptide (micasin). Mutations of its E^8^ by A, R, Q, Leu (L), and K all enhanced its antibacterial activity (Wu et al. 2016). 4) The hormone leptin mutant. Mutations at site D^23^ with A, F, G, H, K, L, R, and W all increased soluble receptor binding affinity and the biological inhibitory potency (Shpilman et al. 2011). 5) ACE2, as mentioned previously (Chan et al. 2020). In view of their presence in these diverse proteins and an unexpected mutational effect, we extended the name of EAMS to Universally Enhanceable Activity-Modulating Site (abbreviated as UEAMS) (fig. 7). Accordingly, we call this approach Paralog-based Discovery of UEAMSs for Saturation Mutagenesis (abbreviated as PD-UEAMS-SM). Because the universal enhancement is only observed at the UEAMS of Crem-5 (i.e., E^15^) but not at the equivalent site of the orthologous Clat-5, we speculate that this mutational behavior is of sequence-dependency due to the difference in their genetic background (epistasis). In this case, specific UEAMSs in Clat-5 could be identified by comparison with its own paralogs.

That the wild-type form of a UEAMS only confers the lowest biologically relevant activity could be a consequence of evolutionary optimization by adaptation given that the lowest activity is sufficient to offer organismal fitness. In theory, protein functions cover both external and internal properties. The former exerts roles by means of interactions with other partners (e.g., proteins, DNA, RNA, and biomembranes) to initiate a biochemical reaction, such as recognition, binding, catalysis, and switching; the latter refers to protein stability, solubility, flexibility, and allostery (Nelson and Cox 2013). Evolution could have shaped proteins with a tradeoff between their external and internal functions by fixing some specific amino acids at certain sites, for example, UEAMSs, to maintain protein homeostasis. In this sense, a UEAMS can be more precisely described as a tradeoff site. Indeed, such tradeoff has been observed between the evolution of new enzymatic specificities (extrinsic function) and loss of the protein’s thermodynamic stability (intrinsic function) in some enzymes (Shoichet et al. 1995; Tokuriki et al. 2008). Therefore, when we see UEAMSs, it suggests that evolution has optimized a collection of properties and we are measuring only one.

For Crem-5, the activity-stability tradeoff may not exist since the functionally enhanced mutant E15K was highly thermal-stable (fig. 4*C*). Further evidence comes from the calculation of relative free energies of folding (supplementary fig. S5, Supplementary Material online), in which SM of Crem-5 at E^15^ resulted in <2 kcal mol^−1^ of energy change (note: ≥+2 kcal mol^−1^ is a threshold of significantly decreased stability; Studer et al. 2013) for all variants, indicative of no structural destabilization. As control, SM of Gly^29^, a well-defined structural residue in all cysteine-stabilized α-helix and β-sheet peptides (e.g., defensins and scorpion toxins) (Bontems et al. 1991; Zhou et al. 2019), adds energy from 6.92 to 56.5 kcal mol^−1^ (supplementary fig. S5, Supplementary Material online). This finding is consistent with the previous observation that directed evolution can create enzymes that are both highly stable and highly active (Arnold et al. 1999). Therefore, an alternative explanation for Crem-5 is that a tradeoff among different activities other than between the activity and stability is evolved if we assume it acts as a multifunctional molecule, just like its homolog (Drs) in *Drosophila* (Cohen et al. 2009; Zhang et al. 2017). In this case, mutations at E^15^ improve its antifungal property but pay a price for other function(s) that we have not known.

Apart from the external functional constraint discussed above, internal epistatic interaction in Crem-5 may exert a role in limiting the evolution of its UEAMS. In comparison with the Crem family, the Clat family evolutionarily loses three members (fig. 6*B*), which likely prompts the evolution of a higher active Clat-5 in *Cae. latens* through the mutation at site 7 to relieve the epistatic control. Given the epistasis prevalence (Breen et al. 2012) and the inherent tradeoff in protein evolution (Pál et al. 2006), we speculate that UEAMSs may prevail in the protein universe implicated in a diversity of biological processes. Once confirmed, our strategy will readily extend to other protein drugs to enhance their activity, consequently lowering their cost and nonspecific cytotoxicity by cutting down the dosage, which will accelerate them into clinical use.

Although further detailed studies are required to answer how UEAMS mutations evoke a universal activity enhancement and why the output does not correlate with amino acid chemistry, as a protein engineering approach PD-UEAMS-SM may be more advantageous than the traditional design approaches based on chemical and structural features of AMPs. These approaches usually need to introduce more mutations for activity improvement (Hilpert et al. 2005; Loose et al. 2006) that might increase the immunogenicity of human-sourced AMPs or therapeutic proteins (De Groot and Scott 2007). In comparison with the general protein engineering tool—neutral drifts-based directed evolution (Peisajovich and Tawfik 2007; Gupta and Tawfik 2008), our approach can rapidly identify target sites with the aid of paralog comparison for SM without the need to construct a neutral drift library (Gupta and Tawfik 2008). Although promising, this approach still has some limitations: 1) If paralogs contain insertion/deletion (indel) mutations and these mutations are relevant to the functional change of a member, as in the case of the scorpion toxin MeuNaTxα-3 (Zhang et al. 2016), our approach may be not applicable since in this case point mutations are not the only one factor responsible for the functional change, lowering the feasibility of this approach. 2) PD-UEAMS-SM requires the functional data known for each paralog for comparison and thus will not applicable for the single-copy gene product or in the absence of enough functional data. However, this will not be a problem when engineering bioactive peptides (e.g., AMPs, peptide neurotoxins, hormones, etc.) as they often exist as multigene families (Kordis and Gubensek 2000; Wu and Brown 2006; Zhu and Gao 2014) and their functions are easily measured using chemically synthesized or recombinant products.

Regardless of these potential limitations, our approach that involves the use of paralog analyses do successfully engineer a cytokinesis-disrupting antifungal defensin and create a collection of enhanced mutants with potential value as antifungal agent leads. Furthermore, it may make a remarkable contribution to understanding the significance of UEAMSs in the evolution and engineering AMPs or other proteins. More studies on UEAMSs will lead to a better understanding of the structural and functional evolution of proteins in the context of tradeoff and epistasis. This also might result in further identification of UEAMSs relevant to other properties of AMPs or other proteins, such as lifespan, solubility, allostery, and toxicity. Given some computational algorithms developed to relate amino acid positions to their specific function and variation-driven functional tuning (Ye et al. 2008; Miller et al. 2019), it is likely that new UEAMSs would be exploited from these positions through site-directed SM.

## Materials and Methods

### Expression Vector Construction and Site-Directed Mutation

To construct the pGEX-4T-1-cremycin-5 expression vector, we used pGM-T-cremycin-5 previously reported (Zhu and Gao 2014) as template for PCR amplification by primers Crem-5-FP and Crem-5-RP containing a *Bam*HI and a *Sal*I restriction site, respectively. To remove the GST tag, codons encoding the enterokinase (EK) cleavage site were introduced between GST and Crem-5 via Crem-5-FP. PCR products were digested by *Bam*HI and *Sal*I and then ligated into pGEX-4T-1. Inverse PCR was used to generate all mutants as previously described (Wu et al. 2016). The gene encoding Clat-5 with EK cleavage was synthesized by BGI-Tech (Beijing, China) with *E. coli* rare codons optimized to improve its expression. The synthesized gene and the mutant Crem-5-E15K/G36A obtained by inverse PCR were individually ligated into pET28a-SUMO through homologous recombination. All primers used are listed in supplementary table S3, Supplementary Material online.

### Peptide Expression, Purification, and Characterization

Recombinant plasmids were transformed into *E. coli* BL21(DE3) host cells grown in LB medium (1% tryptone, 0.5% yeast extract, and 0.5% NaCl, pH 7.2). Expression of fusion protein was induced by 0.1 mM IPTG at an OD_600_ of 0.6. *Escherichia coli* cells were harvested after induction for 4 h at 37 °C by centrifugation and were resuspended in Resuspension buffer (20 mM Tris–HCl, pH 7.5; 150 mM NaCl) for sonication. Fusion proteins were collected by affinity chromatography with glutathione-sepharose 4B beads (GE Healthcare) or Ni-NTA resin (Merck) and digested with EK (Sinobio Biotech Co. Ltd, Shanghai, China) at 25 °C for 4 h. Released proteins were purified by reversed-phase high-pressure liquid chromatography (RP-HPLC) with a C_18_ column (Agilent Zorbax 300SB, 4.6 × 150 mm, 5 μm). Elution was carried out with a linear gradient of 0–60% acetonitrile in 0.05% TFA (v/v) within 40 min with a flow rate of 1 ml/min. To produce ^15^N-labeled protein for NMR, *E. coli* BL21(DE3) cells transformed with the pGEX-4T-1-cremycin-5 plasmid were grown in M9 minimal medium (0.6% Na_2_HPO_4_, 0.3% KH_2_PO_4_, 0.05% NaCl, 0.1% ^15^NH_4_Cl, 0.2% glucose, 0.001% Thiamine, 0.012% MgSO_4_, 0.001% CaCl_2_, and 33 μM FeCl_3_) instead of LB. The method for preparation of ^15^N labeled proteins was the same as described above. Molecular weights of all recombinant peptides were determined by matrix-assisted laser desorption ionization time-of-flight mass spectra (MALDI-TOF MS) on ultrafleXtreme MALDI-TOF/TOF Mass Spectrometer (Bruker Daltonics, Bremen, Germany) in the positive-ion reflection mode and α-cyano-4-hydroxycinnamic acid (CHCA) as a liquid matrix.

### NMR Structural Analysis

The peptide was dissolved in D_2_O or H_2_O containing 10% D_2_O at pH 4.6. All NMR experiments at 298.0 K were performed on a Bruker AVANCEIII 800, the ^1^H resonance frequency of 800.23 MHz, equipped with a TCI cryogenic probe (Bruker Biospin). The obtained NMR data were processed with NMRPipe (Delaglio et al. 1995) and analyzed with Sparky (http://www.cgl.ucsf.edu/home/sparky/, last accessed February 23, 2017). The three-dimensional structure was calculated with XPLOR-NIH (Schwieters et al. 2003). The NMR-derived structure was subjected to molecular dynamic simulation with myPresto (Gil Kim et al. 2003).

### Circular Dichroism Spectroscopy

Circular dichroism (CD) spectra of all peptides were recorded on Chirascan^TM^-plus CD spectrometer (Applied Photophysics Ltd, United Kingdom) at room temperature from 180 to 260 nm with a quartz cell of 1.0 mm pathlength. Spectra were measured at a peptide concentration of 0.1–0.15 mg/ml in water. Data were collected at 1 nm intervals with a scan rate of 60 nm/min and expressed as delta epsilon (cm^−1^M^−1^) calculated as [*θ*×(MRW × 0.1)/(*C* × *L*)/3,298], where *θ* is the ellipticity (in millidegrees), *C* is the concentration (in mg/ml), *L* is the pathlength (in cm), and MRW is the mean residue weight (in Da).

### Antifungal Assay

Lethal concentration (*C*_L_) of a peptide was determined by the inhibition zone assay performed according to the method previously reported (Hultmark 1998; Ekengren and Hultmark 1999). Briefly, filamentous fungi were incubated on potato dextrose agar (PDA) (20% potato, 2% glucose, and 1.5% agar) at 30 °C for 1 week. Spores were harvested and suspended in sterile water with an OD_600_ of 0.5. Six ml of PDA containing 0.8% agar was mixed with 50 μl spores suspension and poured into Petri dishes of 9.0 cm diameter, giving an agar depth of 1 mm. Wells with a diameter of 2 mm were punched into the medium, filled with 2 μl of twofold serially diluted peptides at three different doses. Plates were incubated at 30 °C overnight and zones of inhibition were measured. *Candida albicans* grown in potato dextrose broth (PDB) (20% potato, 2% glucose) at an OD_600_ of 0.5 were used as described above. Diameters of inhibition zones were used to calculate *C*_L_ of each peptide. The squared diameter (in cm) of the inhibition zone is plotted against the log amount of peptide (in nanomoles), and *C*_L_ is calculated in micromolar from the slope (*k*), the intercept (*m*), and the agar depth (*a*, in cm) by using a diffusion equation according to the following formula: *C*_L_ = 2.93/(*ak*10^*m*^^/^^*k*^).

MICs were measured based on broth microdilution method (CLSI 2008). *Candida albicans* B16 cells were inoculated into PDB at 30 °C overnight. The culture was diluted down to 1 × 10^3^ colony-forming units (cfu)/ml and a peptide was added to each culture with a final concentration of 0.78, 1.56, 3.13, 6.25, 12.5, 25, and 50 μM. Followed by incubation at 30 °C for 24 h, cultures were streaked on a PDA plate to assess the viability of *C. albicans* B16. The lowest concentration in the series at which no growth is observed is recorded as MIC. The source of fungi used in this assay is provided in table supplementary S4, Supplementary Material online.

### Killing Kinetics Assay

The in vitro killing curve for E15K was determined based on the previous procedure (Boman et al. 1993). 5×*C*_L_ of peptides were added to *C. albicans* B16 culture (1 × 10^6^ cfu/ml). Samples were taken, diluted, and plated after incubation of 0.5, 1, 1.5, 2, 3, 4, 5, and 6 h. Medium and 5×*C*_L_ of clotrimazole (Sigma) and amphotericin B (Amersco) were used as negative and positive control, respectively.

### Hemolytic Assay

Hemolysis of mouse erythrocytes by peptides was assayed according to the standard method (Gao et al. 2009). In brief, the cells were incubated with various concentrations of peptides (6.25, 12.5, 25, or 50 μM) at 37 °C for 15 min. Absorbance was measured at 570 nm with the SpectraMax i3x Multi-Mode microplate reader (Molecular Devices). The percentage of hemolysis was determined as (*A*_pep_−*A*_blank_)/(*A*_tot_−*A*_blank_)×100. *A*_blank_ and *A*_pep_ were evaluated in the absence or presence of peptides and 100% hemolysis (*A*_tot_) was obtained in the presence of 1% Triton X-100. Meucin-18 (Gao et al. 2009) was used positive control.

### Peptide Stability Assay

To assess serum stability, peptides were incubated in fresh mouse serum for 0, 12, and 24 h at 37 °C, respectively. Residual activities were measured by inhibition zone assays against *C. albicans* B16. For thermal stability assay, E15K was incubated at 50 °C for 1–5 days, 80 °C or 100 °C for 5 min. CD analysis and inhibition zone assay were used to evaluate a potential impact of heating on the structure and function of the peptide.

### Scanning Electron Microscopic Observation

*Candida albicans* B16 cells were treated with E15K at 5×*C*_L_ at 30 °C for 6 h and were fixed with 2.5% glutaraldehyde for 1 h, followed by washing three times with PBS. Cells were dehydrated with a series of graded ethanol solution and then dried by a critical point dryer (Leica EM CPD300, Austria) before being mounted on carbon tape, sputtered with gold coating (Leica EM SCD050, Austria). Images were visualized by a SEM (FEI QUANTA 450, Czekh).

### Molecular Dynamics Simulations

Using the MUTATE function of the Swiss PDB Viewer (http://spdbv.vital-it.ch/, last accessed July 2, 2017), we constructed the 3D structures of all E^15^ mutants based on the average NMR-based structure of Crem-5 determined here, which were used in MD simulations.40ns of MD simulations were performed for each structure by Gromacs 5.1.4. (http://www.gromacs.org/, last accessed July 2, 2017) with all-atom OPLS force field to create Gromacs topology (Jorgensen et al. 1996; Kaminski et al. 2001). For MD simulations, the structure was immersed in a cubic box extending at least 1 nm from the protein surface. The system was solved with simple point charge (SPC) water (Berendsen et al. 1987) and neutralized with Na^+^ and Cl^−^ ions. Energy minimization was done with the steepest descent algorithm for 5,000 steps to remove large forces. NVT (number of particles, volume, and temperature) and NPT (Number of particles, pressure, and temperature) ensembles at 300 K were sequentially used to generate the initial equilibrated structure. All bond lengths were constrained with the linear constraint solver (LINCS) algorithm (Hess et al. 1997) and trajectories were saved every 10 ps. Cα RMSD and Cα root-mean-square-fluctuation (RMSF) were calculated after simulations. The last MD simulation frame was used to analyze H-bonds by LigPlot^+^ software (Laskowski and Swindells 2011) (http://www.ebi.ac.uk/thornton-srv/software/LigPlus/, last accessed October 26, 2017).

### Statistics

Data are expressed as mean ± SD from three independent experiments. Unpaired two-tailed Student’s *t*-test was used to compare means between control and treatment group with SPSS (SPSS Inc.). Generally, *P *<* *0.05 (*) was considered statistically significant, *P *<* *0.01 (**) was considered highly significant and *P *<* *0.001 (***) was considered very highly significant. 

## Supplementary Material

Supplementary data are available at *Molecular Biology and Evolution* online.

## Supplementary Material

msab224_Supplementary_Data
