# Supplementary material for: Evolution-Based Protein Engineering for Antifungal Peptide Improvement
Source: Mol Biol Evol. 2021 Jul 28;38(11):5175–89. doi: 10.1093/molbev/msab224 (PMC8557468; doi:10.1093/molbev/msab224)
Supplement: msab224_Supplementary_Data [file msab224_supplementary_data.pdf]

Supplementary Material

Evolution-Based Protein Engineering for Antifungal Peptide Improvement

Jing Gu, Noriyoshi Isozumi, Shouli Yuan, Ling Jin, Bin Gao, Shinya Ohki, and Shunyi Zhu

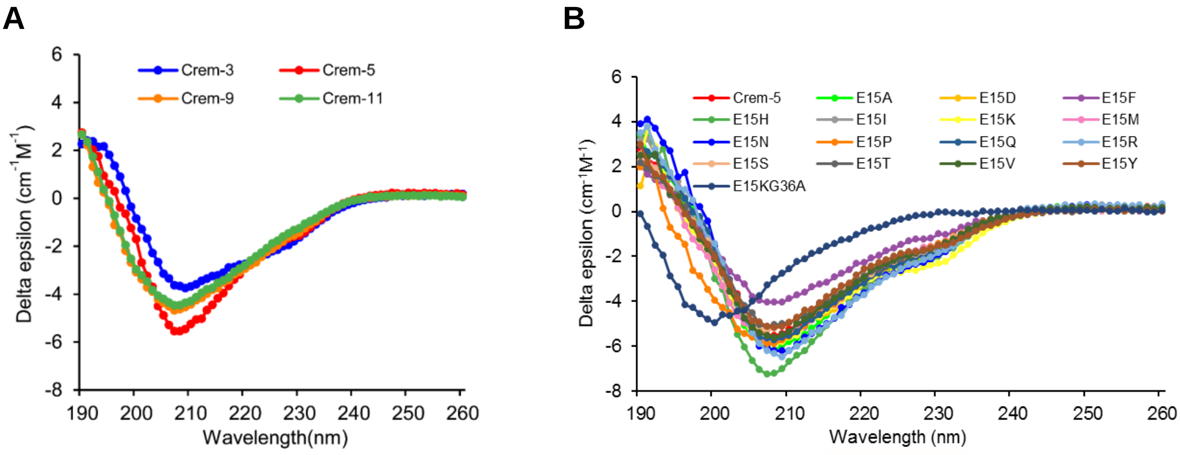

FIG. S1. CD spectroscopy of recombinant peptides. (**A**) Crem-5 and its paralogs. (**B**) Crem-5 and its E15 mutants.

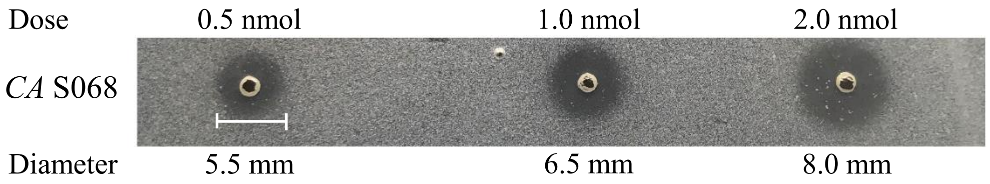

FIG. S2. Inhibition-zone assay for assessing the antifungal activity of E15N (as a representative). Three different doses (0.5, 1.0 and 2.0 nmol) were used to produce dose-dependent inhibition zones in the *CA* S068 agar plate for C_L determination.

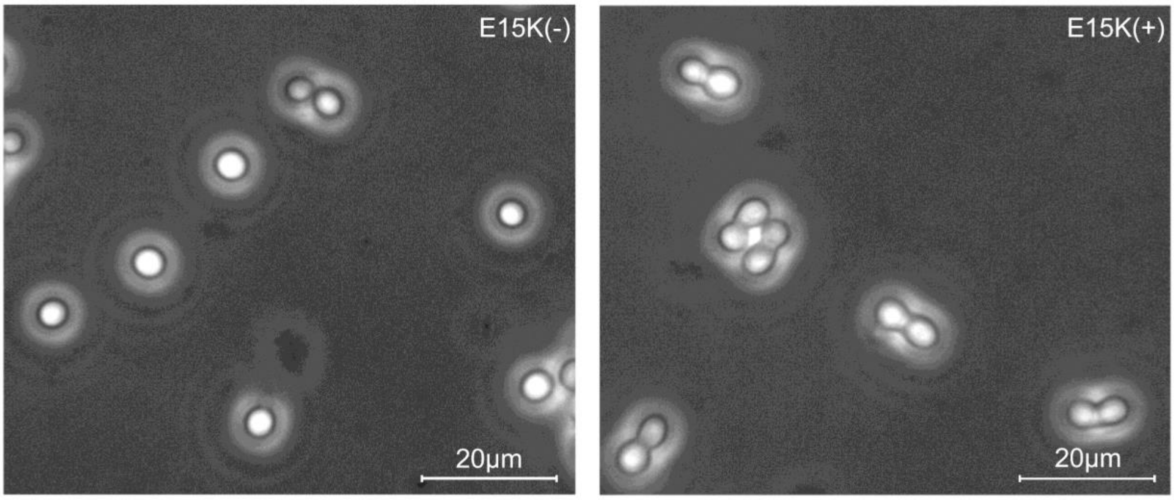

FIG. S3. Phase contrast microscope observation of the effect of E15K on *C. albicans* B16. Freshly cultured cells were treated by 5×C_L E15K and observed by the microscope Axio Observer A1 (Carl Zeiss Jena, Germany) after 6 h of incubation at 30°C. "+" and "-" indicate the presence and absence of E15K, respectively.

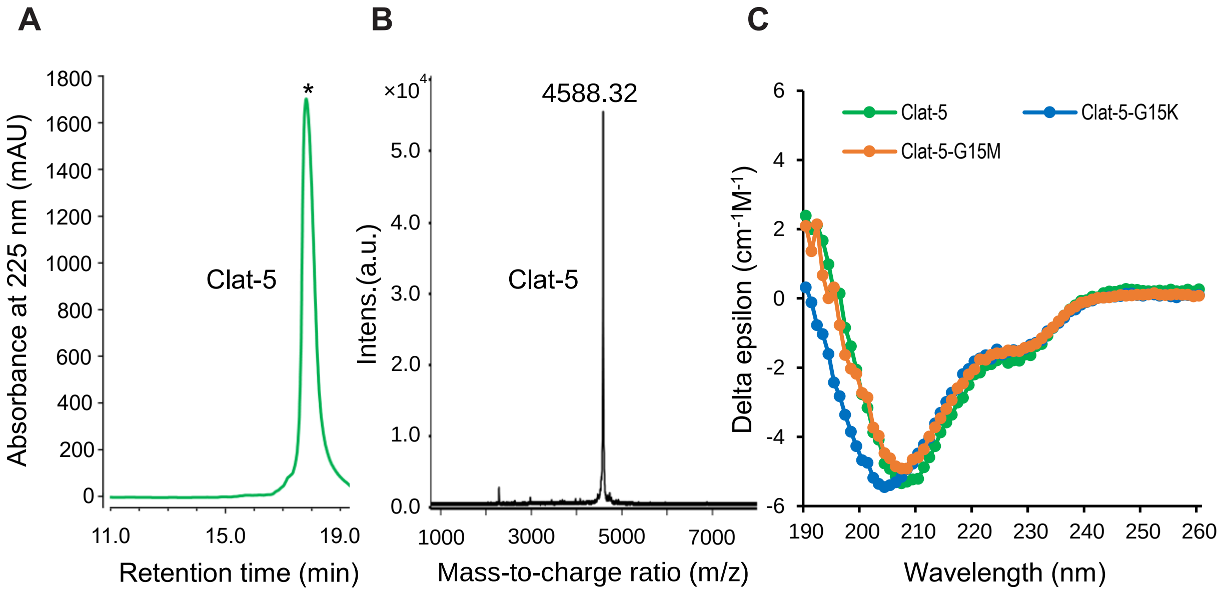

FIG. S4. Expression, purification and characterization of Clat-5 and its mutants. (**A**) RP-HPLC profiles recombinant peptides (Clat-5 as a representative). The product collected for analysis is marked by an asterisk. (**B**) MALDI-TOF MS of HPLC-purified recombinant Clat-5. (**C**) Comparison of CD spectra between Clat-5 and its G^{15} mutants.

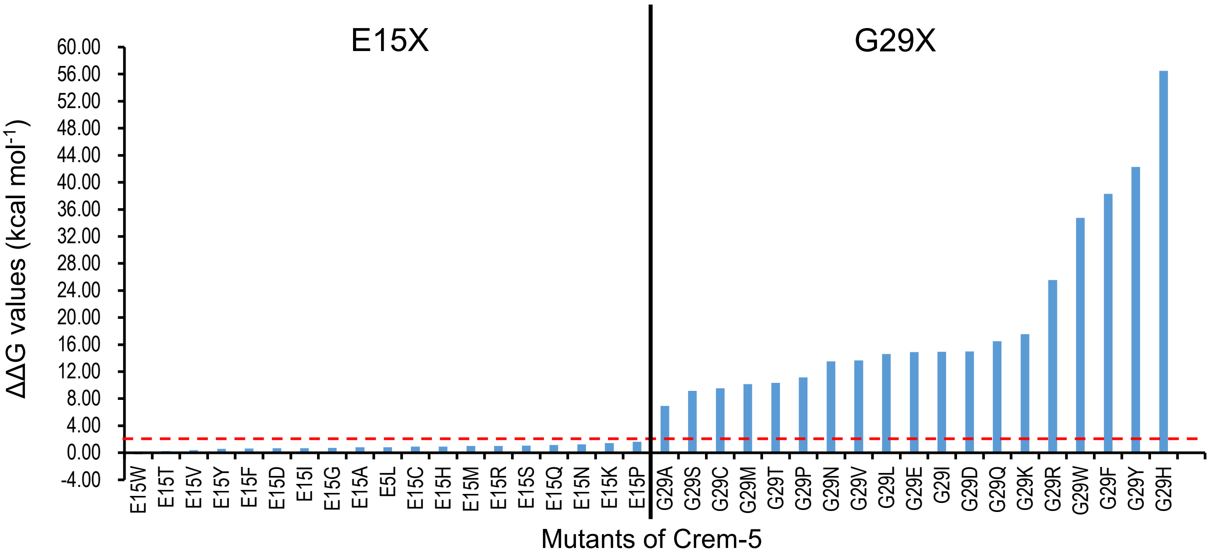

FIG. S5. The relative free energies of folding (ΔΔG) for Crem-5 mutants. The stabilities of Crem-5 and its mutants were predicted by FoldX version 4.0 (http://foldx.embl.de/). Mutations were performed with the <PositionScan> command and all options were set to default. The ΔG is the difference in free energy between the unfolded and folded state of the protein ($\Delta G = G_{folded} - G_{unfolded}$). The ΔΔG is the difference of unfolding Gibbs free energy (ΔΔG) between mutant and WT ($\Delta\Delta G = \Delta G_{mutant} - \Delta G_{WT}$), which is used to calculate how much a protein mutation affects the stability. ΔΔG < +2, marked by a red dotted line, represents the threshold of significantly decreased stability.

Table S1. Molecular weight of recombinant peptides

Peptides	Experimental MW (Da)	Theoretical MW (Da)
Crem-5	4674.81	4676.08
K3A	4619.24	4618.98
H6A	4608.80	4610.02
H13A	4609.27	4610.02
E15A	4617.47	4618.04
E15C	not designed	
E15D	4660.80	4662.05
E15F	4691.31	4694.14
E15G	formation of insoluble inclusion bodies	
E15H	4683.07	4684.11
E15I	4659.20	4660.12
E15K	4673.58	4675.14
E15KG36A	4689.42	4689.17
E15L	non-specific cleavage of fusion proteins	
E15M	4676.31	4678.16
E15N	4660.96	4661.07
E15P	4643.21	4644.08
E15Q	4674.80	4675.09
E15R	4702.81	4703.15
E15S	4631.20	4634.04
E15T	4646.66	4648.07
E15V	4644.10	4646.10
E15W	formation of insoluble inclusion bodies	
E15Y	4708.10	4710.14
R33A	4590.50	4590.97
Clat-5	4588.32	4588.02
Clat-5-G15K	4659.23	4659.14
Clat-5-G15M	4661.75	4662.16

Note: Molecular weights of the recombinant peptides were determined by MALDI-TOF MS.

Table S2. Lethal concentrations (C_L) of Crem-5 and its mutants against fungi

	C_L (μM)		
	A. nidulans A28	*A. fumigatus YJ-407*	*A. niger*
Crem-5	W.A.	15.45	4.03
K3A	N.A.	N.A.	W.A.
H6A	W.A.	N.A.	W.A.
H13A	W.A.	W.A.	W.A.
E15A	7.25	7.70	2.74
R33A	W.A.	N.A.	W.A.

Note: "W.A.", weak activity, indicating that only very small inhibition zones were observed at 1.6 nmol peptide/well; "N.A.", no activity, indicating that no inhibition zones were observed at 1.6 nmol peptide/well.

Table S3. PCR primers used in this study

Name	Sequence (5'→3')
Crem-5-FP	ATGGATCCGATGACGATGACAAGGATGTCAAAAGTGGACAC
Crem-5-RP	ATGTCGAC*TTA*TGTGTCACACCAACAAGC
K3A-FP	**GCA**AGTGGACACTACAAAGGACCA
K3A/H6A-RP*	GACATCCTTGTCATCGTCATCGGA
H6A-FP	AAAAGTGGA**GCC**TACAAAGGACCA
H13A-FP	**GCT**GACGAGAATTGTAATGGCGTT
H13A/E15X-RP*	GTAGCATGGTCCTTTGTAGTGTCC
E15A-FP	CATGAC**GCG**AATTGTAATGGCGTT
E15D-FP	CATGAC**GAT**AATTGTAATGGCGTT
E15F-FP	CATGAC**TTC**AATTGTAATGGCGTT
E15G-FP	CATGAC**GGG**AATTGTAATGGCGTT
E15H-FP	CATGAC**CAC**AATTGTAATGGCGTT
E15I-FP	CATGAC**ATT**AATTGTAATGGCGTT
E15K-FP	CATGAC**AAA**AATTGTAATGGCGTT
E15KG36A-FP	CGTTGGGGA**GCT**GCTTGTTGGTGT
E15L-FP	CATGAC**CTG**AATTGTAATGGCGTT
E15M-FP	CATGAC**ATG**AATTGTAATGGCGTT
E15N-FP	CATGAC**AAC**AATTGTAATGGCGTT
E15P-FP	CATGAC**CCG**AATTGTAATGGCGTT
E15Q-FP	CATGAC**CAA**AATTGTAATGGCGTT
E15R-FP	CATGAC**CGT**AATTGTAATGGCGTT
E15S-FP	CATGAC**AGT**AATTGTAATGGCGTT
E15T-FP	CATGAC**ACG**AATTGTAATGGCGTT
E15V-FP	CATGAC**GTG**AATTGTAATGGCGTT
E15W-FP	CATGAC**TGG**AATTGTAATGGCGTT
E15Y-FP	CATGAC**TAC**AATTGTAATGGCGTT
R33A-FP	**GCT**TGGGGAGGAGCTTGTTGGTGT
R33A/E15KG36A-RP*	GCTGCAGTGACCAGATTTGTAGCC
Clat-5-SUMO-FP	ACAGAGAACAGATTGGTGGAGACGACGACGACAAAGAC
Clat-5-SUMO-RP	AGTGCGGCCGCAAGCTTG*TTA*GGTGTCGCACCAGCA
E15KG36A-SUMO-FP	ACAGAGAACAGATTGGTGGAGATGACGATGACAAGGAT
E15KG36A-SUMO-RP	AGTGCGGCCGCAAGCTTG*TTA*TGTGTCACACCAACA

Note: Restriction endonuclease sites (*Bam* HI and *Sal* I) and mutated nucleotides are underlined and boldfaced, respectively; and the stop codon is italicized. "*", reverse primers used to combine with respective forward primers to obtain corresponding mutants. Homologous arms are shadowed in grey.

Table S4. Fungi used in this study

Fungus	Isolation site	Source
Aspergillus niger	Polyvinyl chloride (PVC) plastic	Center for Microbial Resources, Institute of Microbiology, Beijing, China
Aspergillus nidulans A28	X-ray mutant of A4 → UV mutant of A26	Prof. Shaojie Li, Institute of Microbiology, Beijing, China
Aspergillus fumigatus YJ-407	Soil	Prof. Cheng Jin, Institute of Microbiology, Beijing, China
Candida albicans 2.4138	Pleural effusion from gastric cancer patient	Prof. Fengyan Bai, Institute of Microbiology, Beijing, China
Candida albicans B16	Vagina of Chinese women suffering from VVC	Prof. Fengyan Bai, Institute of Microbiology, Beijing, China
Candida albicans GD18	Sputum	Prof. Fengyan Bai, Institute of Microbiology, Beijing, China
Candida albicans S068	Vagina from asymptomatic women	Prof. Fengyan Bai, Institute of Microbiology, Beijing, China

Table S5. Input NMR data and structural statistics for the final 20 structures of Crem-5

**Number of experimental restraints**	
Total Number of NOEs	135
Short-range | i - j | ≤ 1	56
Medium range 1 < | i - j | < 5	24
Long range 5 ≤ | i - j |	25
Hydrogen bond restraints	30
**Structure statistics, 20 conformers**	
Average pairwise r.m.s.d.(Å)	
Backbone atoms (residues 1-29)	1.00 ± 0.37
Heavy atoms (residues 1-29)	1.64 ± 0.37
PROCHECK Ramachandran plot analysis (after the MD refinement)	
Residues in favored regions (%)	66.0
Residues in additionally allowed regions (%)	26.0
Residues in generously allowed regions (%)	7.0
Residues in disallowed regions (%)	0.0

Table S6. The accession numbers, origins, molecular sizes and masses of clatencins

Name	Accession No. (position)	Origin	Size	Molecular mass (Da)
Clat-1	NIPN01000032.1 (532630-532886)	*C. latens* PX534 scaffold31	42	4524.06
Clat-3	NIPN01000032.1 (534966-535204)	*C. latens* PX534 scaffold31	42	4495.92
Clat-4	NIPN01000032.1 (532361-532001)	*C. latens* PX534 scaffold31	42	4640.09
Clat-5[a]	NIPN01000032.1 (535729-535491)	*C. latens* PX534 scaffold31	42	4588.02
Clat-6	NIPN01000032.1 (536021-#)	*C. latens* PX534 scaffold31	—	—
Clat-7[a]	NIPN01000134.1 (190128-189891)	*C. latens* PX534 scaffold133	42	4546.98
Clat-8[a]	NIPN01000134.1 (190536-190773)	*C. latens* PX534 scaffold133	42	4546.98
Clat-9	NIPN01000032.1 (534674-#)	*C. latens* PX534 scaffold31	—	—
Clat-10[a]	NIPN01000032.1 (536966-536728)	*C. latens* PX534 scaffold31	42	4541.95
Clat-12	NIPN01000032.1 (#-531650)	*C. latens* PX534 scaffold31	42	4497.94
Clat-13[a]	NIPN01000032.1 (537344-537582)	*C. latens* PX534 scaffold31	42	4554.01
Clat-15	NIPN01000032.1 (538247-537996)	*C. latens* PX534 scaffold31	40	4606.30
Clat-10φ	NIPN01000032.1 (533729-533496)	*C. latens* PX534 scaffold31	42	—

Note: “#” represents positions undetermined in this study; “—” indicates data not available due to incomplete sequence or pseudogene (φ). [a] Proteins currently annotated in the GenBank database as hypothetical proteins.
